# Diseases of the Chest

**Published:** 1838-01-01

**Authors:** 


### DISEASES OF THE CHEST AND WINDPIPE.

### I. DISEASES OF THE CHEST.

A Treatise on the Diagnosis and Treatment of Diseases of the Chest. Part I. Diseases of the Lung and Windpipe. By *William Stokes*, M.D. M.R.I.A., Dublin. Hodges and Smith.

On glancing at the first page of the Preface of this book we were not a little gratified to find the author state that, in the composition of it, he has made it a principal object to connect the study of physical signs with that of symptoms, "so as to illustrate their mutual bearing on diagnosis, and remove that unjust opprobrium thrown on the advocates of auscultation, that they neglect the study of symptoms." That there may be some ground for this charge of a neglect of symptoms by some persons professing themselves followers of the modern pathology, may be true. It requires some caution to avoid extremes. In endeavouring to steer clear of the Scylla of mere symptomatology on the one hand, some wrecks may have taken place by striking against the Charybdis of sheer physical semeiology on the other.

Those, however, who make such a charge of neglect of symptoms against the educated followers of modern pathology, we refer to the writings of the Choryphæus of modern pathology, Andral. They will there find that rational symptomatology has been any thing but neglected. There the study of symptoms will be found to have received ample attention, and to have been pursued with zealous care; in that way, however, in which alone advantage could be rationally expected from it, namely, in connexion with physical signs, wherever this was possible. The individuals, however, who make these charges against the cultivators of modern pathology, and those particularly who make a practice of abusing one implement of it, we mean the stethoscope, are irreclaimable. They are either too old, too lazy, or too stupid to learn. Happily, however, for the rising generation, their ranks are thinning every day—for this a variety of causes may be assigned. Some of them have gone the way of all flesh. Some, convinced of the utility of stethoscopic investigation, have actually and in right down earnest set themselves down, and learned the use of the instrument—while others, again, without the industry, or probably without the physical power to learn it, affect to understand it, and really carry it about with them, though we know some of them, who would just derive as much benefit from applying it to the seat of honour as to the chest. Such, however, is the effect of the enlightened state of public opinion of the present day, which will no longer tolerate the mere symptomatic doctor of the last century. Something more than an acquaintance with a Latin and Greek Vocabulary, some other badge of professional knowledge beside the wig and gold-headed cane, is now required from the medical candidate for public confidence. We would venture a small wager that that so much abused, insignificant-looking little instrument, the stethoscope, would prove a surer passport in the hands of a young medical aspirant for public employment, and a more certain means of obtaining public confidence as a physician, than the gold-headed cane of Sydenham, Meade, or of any other of the medical sages of former times.

But to come to our more immediate business. Our author devotes the first section of his book to the exposition of the *General Principles of the Diagnosis of Thoracic Disease.* There is so much of force and truth in the following remark of Dr. Stokes, that we cannot but cite it in his own words:

"It must be obvious, that in the detection of the nature and seat of any disease, the more we can combine the observation of physical signs with functional symptoms, the greater will be the accuracy of our diagnosis. Now, if we compare together the diseases of the three great splanchnic cavities, we find that those in which this desirable combination is most attainable are, first, those of the chest; next, the abdomen; and lastly, the affections of the brain and spinal marrow. Accordingly, if we compare the diseases of these systems with respect to the perfection of diagnosis, we find the order to be, first, the respiratory; next the abdominal; and last the cerebro-spinal, or that in which the combination is least applicable." 3.

Our author now shows that the contents of the chest in the healthy and diseased state are most favorably circumstanced for the multiplication and distinctness of physical signs; to which circumstance he traces the great improvements by which the present time is distinguished in the diagnosis and consequently the treatment of thoracic disease. This he proves from the consideration of the anatomical structure, and also the functions of the thoracic organs, as compared with the contents of the other cavities. Among the other cogent and unanswerable arguments urged by our author in support of the advantage of physical examination, he appeals to the frequency of the latent affections of the lung, as showing the necessity of this mode of investigation. The lung, we know, may be hepatized without cough, dyspnœa, acceleration of breathing, pain, expectoration, or fever. But this change cannot occur without the existence of physical signs sufficient for its detection.

"It is plain," says the author, "that the study of symptoms alone cannot lead to accurate distinction of chest disease; the same remark is applicable to that of the physical signs unconnected with symptoms. Symptoms are insufficient without signs, and signs insufficient without a careful comparison of these with the symptoms. There is no such thing as a perfectly pathognomonic symptom or sign of any such thoracic disease. We must combine the lights drawn from the careful study of symptoms, both past and present, with the observation of physical signs, for by this mode alone, can we hope to arrive at an accurate result. Great injury has been done to the cause of physical diagnosis by some inexperienced men, who, departing from the principles of its illustrious founder, have neglected too much the study of symptoms."

After enumerating the sources of physical diagnosis, namely—1st. Signs purely acoustic, including the results of percussion and auscultation. 2nd. *Signs* derived from the alterations of shape and volume of the thorax. 3rd. *Signs* referrible to the sense of touch. 4th. *Signs* derived from inspecting the motions of the thorax during respiration. 5th. *Signs* derived from inspection of the thorax with reference to the action of the heart and great vessels. 6th. *Signs* derived from the existence of an external collateral circulation. 7th. *Signs* derived from the observation of the displacement of the thoracic or abdominal viscera. He next proceeds to elucidate the principle of *comparison.*

He adduces the following as an important and common illustration of the value of comparison.

"A patient presents the symptoms of cough, muco-purulent expectoration, accelerated breathing and pulse, emaciation, and hectic. Under these circumstances, we detect a mucous rattle in the subclavicular region; a sign which, when properly estimated and corrected, may lead to an almost positive diagnosis of phthisis, with softening of the tubercles. Now, if, in a patient labouring under the above symptoms, we were to conclude from the mere existence of this sign in this situation, that the case was really phthisis, we might fall into error, for a comparative examination of the different portions of the chest might shew, that the rule was universal; a discovery, which would greatly diminish its value as a sign of phthisis, and leave a probability that the case was one of bronchitis, with copious effusion into the smaller tubes. In such a case the value of comparison is obvious."

"On the other hand, the existence of râle, either under one or both clavicles, while the inferior portions remained free, would, when occurring with the symptoms described, be a most important diagnostic of phthisis."

Our author having in the first section considered the general principles to be observed in the diagnosis of thoracic disease, now proceeds to treat of the individual diseases of the lung, and commences with *bronchitis*, the study of which is peculiarly calculated to furnish us with a key to thoracic pathology, as in a great number of pulmonary, and even cardiac diseases, the inflammation of the mucous membrane of the lung seems to be the first link in the chain of morbid action; a circumstance illustrative of the proposition of Broussais, that the various external morbid influences which affect the system are first exercised on one of the surfaces of relation, viz. the skin, the bronchial, and the gastro-intestinal mucous membrane. The importance of studying bronchial inflammation our author endeavours to prove by showing that many examples of diseases, which have received a separate name, have commenced with bronchitis, or are complicated with it; that it is also a prominent feature in certain nervous affections of the lung, that it may occasion dilatation of the air-cells and tubes, and pulmonary emphysema, and that many cases of phthisis also commence by it. The importance of this study is still further established by the circumstance that bronchitis is the most common result of the sympathetic irritations of the lung, that it forms a most important part of the phenomena of several of the exanthemata, whilst in common continued fever it is extremely frequent, and too often the direct cause of a fatal termination.

Having shewn that all ages are liable to this disease, that it is sometimes even congenital, he next alludes to the researches of Dr. Joerg, of Leipsig, on a condition of the lung which that physician states may be induced by either a too rapid, or a too slow delivery; under which circumstances a portion of the lung remains uninflated, which occasions imperfect respiration, and various pulmonary diseases. In a difficult delivery, he states, that the infant, from the compression of the brain, respires imperfectly, and consequently but partially expands its lungs; while in the too speedy delivery, in consequence of its short duration, and the inferior degree of compression of the placenta, the foramen ovale, according to him, is not closed, which diminishes the necessity for respiration.

"Under the circumstances above-mentioned," says Dr. Joerg, "we have often seen infants suddenly seized with illness, and sometimes die, in spite of every exertion made to save them, before the real cause of the attack, and the proper method of treatment, were discovered; and on examination the following appearances were observed, arising all from the same causes, though differing greatly among themselves in many respects.

In every case in which we made a *post-mortem* examination for several years past, a portion only of the lungs, from the greater half to merely an eighth or tenth part, was found filled with air, and of a red colour; while the remaining portion continued in the same state in which it had been in the fœtus, and was of a liver colour. When the infant had died soon after birth, the condensed portion was susceptible of inflation; but where death did not occur till several weeks after that event, it was found carnified and incapable of being inflated; sometimes the partition between the healthy and diseased portion was in a state of inflammation, and the latter contained vomicæ: the bronchi, too, were often inflamed and filled with mucus. The great contrast between the bright red of the healthy, and the liver-brown of the diseased portions, struck the eye immediately on opening the thorax. In most cases the foramen ovale was still open, and there were very firm polypi in the heart and large vessels. The brain was frequently gorged with blood, which was sometimes even effused between its membranes and over its surface: it also occasionally contained abscesses corresponding to others on the cranium, or fontanelle, that had been produced by the use of instruments, or by violent pressure against the pelvis during delivery. In the rest of the body there was no particular morbid phenomenon constantly present: however, in the greater number of cases, the skin, particularly on the face, had a blueish cast: while in some it was withered and emaciated, and the whole body, especially the intestines, pale and bloodless."

Dr. Joerg feels warranted by his experience and observation in describing the nature and terminations of this disease in the following manner. The following extract from this physician's work has been presented to us by the author:—

" The solidification, or continuation in the fœtal condition of a greater or less portion of the lungs, so that during inspiration their substance cannot be penetrated by the air. The blood, being still more incapable of penetrating, cannot be supplied with oxygen, and must consequently continue venous, and produce obstructions and dangerous congestions; while at the same time, from its being unable to afford the stimulus requisite to the system for the continuation of its functions, an atonic senile condition obtains, attended with the utmost weakness, and complete atrophy, and terminating in death in hectic fever. The general morbid condition is, consequently, difficulty of respiration, and impeded circulation, producing dangerous and even fatal congestions. Its terminations are—1st, recovery; 2d, secondary diseases; 3d, death.

I. Recovery ensues when the efforts of the infant to inspire are assisted by proper treatment, and the subsequent symptoms properly managed.

II. *Secondary Diseases.*—*(a)* Obstruction of the lungs, inasmuch as a portion of them remains condensed, which, without actually producing death, is very oppressive and dangerous: *(b)* chronic cyanosis, the foramen ovale continuing open, and the infant being liable to constant suffering.

III. *Death.* *(a)* From apoplexy; in consequence of obstruction and congestion: *(b)* from suffocative catarrh, when the feeble respiration is not able to expel the mucus secreted in the bronchi, and the violent efforts at full inspiration produce bronchitis, and an over-abundant secretion of mucus, which the patient has not strength to get rid of: *(c)* from fever, the result of bronchitis: *(d)* from atrophy; the production of animal heat being prevented by the deficiency of oxygen, and the whole system paralysed by the want of its requisite stimulus.

SYMPTOMS. When the infant comes into the world, the head is either found greatly swollen, (in which case abscesses often form in the part that has suffered from pressure, and inflammation or violent congestion of the brain ensues,) or else, though quite uninjured, and the delivery having been rapid and easy, it cries but feebly, breathes very short, and exerts the muscles of the thorax greatly; it is presently attacked with a faintness, and if it had been capable of drinking previously, now loses that power, the voice becomes hoarse and weak, and scarcely audible. Stertor and convulsions soon follow, the little patient becomes quite blue, the eye-balls turn, and the respiration remits, sometimes for so long as five minutes, till the scene at last closes with death. Should the illness continue for some days or weeks, a little short cough, the most certain sign of violent bronchitis, comes on; together with total weakness, atrophy, and hectic fever; and the child, at the very latest, four or five weeks after birth, sinks under a violent attack of cyanosis, or bronchitis, or from the effects of the fever and atrophy."

This non-expansion of a portion of the lung was not, we believe, noticed by any writer before Dr. Joerg, as the cause of bronchitis in the infant. It serves to afford an explanation of those cases in youth and adult age, in which the patient is represented to have had a cough from his birth; many of these cases terminate in dilatation of the air-cells and emphysema of the lung.

Another cause of bronchial inflammation in the infant after birth is the period of the first dentition—in this case, however, our author very properly remarks that it is rather the result of the general constitutional disturbance, than a primary disease. The presence of this affection is easily recognized; it is generally marked by great irritability, hurried breathing, some wheezing in the throat, and acceleration of the pulse. When the attack is more severe, there is fever and cough, dilatation of the nares during inspiration, and obvious difficulty in sucking. The mouth also is hot, the gums dry and swollen, and one or two teeth are observed to be coming forward. These symptoms continue from four to five days, and often subside rapidly on the appearance of a tooth.

Our author now proceeds to examine the occurrence of bronchitis in the adult. The cases of bronchitis he divides into the primary, secondary, and complicated forms: the primary, those in which the first morbid influence seems to be exercised on the respiratory mucous membrane, and in which the fever, if it exists, may be considered as purely symptomatic: in the secondary, on the other hand, there has been a pre-existing disease elsewhere, which may be either the irritation of another organ, which acts by sympathy on the lung, or that general morbid state commonly called essential fever. By the complicated form our author means the bronchial inflammation which accompanies other diseases of the lung, such as pneumonia, pleurisy, pulmonary apoplexy, tubercle, &c.

Acute Primary Bronchitis. This affection is met with under various conditions. It not unfrequently occurs as a mild, and often a pyrexial disease, in which the irritation seems to be consecutive to an affection of the lining membrane of the nares and throat. One of the most curious symptoms connected with this form of the disease is the tickling sensation perceived in the trachea, which commonly precedes, and seems to be the cause of cough, and which is referred either to the situation of the bifurcation of the trachea, or the portion of the windpipe immediately above it.

From a consideration of the symptoms and the stethoscopic phenomena in this disease, our author is inclined to think, that in the majority of cases the smaller bronchial ramifications are unaffected—fever is either entirely absent, or very slight—there is never any perceptible lividity of countenance —there is scarcely ever any dropsical effusions, a circumstance proving that the pulmonary circulation is not seriously interfered with.

When the disease however occurs in the more severe form, all these symptoms are aggravated; there may be high fever, great dyspnœa, difficult expectoration, the mucus being sometimes tinged with blood; cerebral and abdominal congestions, with dropsical effusions; pneumonia, and in some cases, pleuritis.

After the acute stage has thus continued for a period, which is very variable, the second stage sets in, which is marked by a change in the character of the fever, the inflammatory passing into the hectic type; the countenance becoming pale and shrunk, and the pulse feeble and rapid. The patient perspires; the cough is frequent though less distressing, and is followed by copious expectoration of concocted mucus or muco-purulent matter, and the breathing, though hurried, is generally less laborious than in the acute stage. A person unacquainted with the history of the case, would suppose the patient to be in an advanced stage of suppurative phthisis. Often, as our author observes, has the recovery of such an individual been set down as an instance of the cure of confirmed phthisis.

The appearance of the expectoration in acute bronchitis he thus describes: "In the earlier forms the secretion is scanty, and consisting of a clear gelatinous mucus, combined with a frothy serum: according as the disease advances this secretion becomes more opaque, more abundant, and less tenacious; and at that period when the inflammatory fever ceases, and is either succeeded by an apyrexial state, or by a hectic condition, we observe a remarkable change in its character. It becomes thick and has considerable consistence, or it may pass into the muco-puriform character, when we observe it in masses of a greenish-yellow colour, quite opaque, and though somewhat viscid, yet flowing together." After observing on the comparative rarity of the more violent primary bronchitis in adults, though a common occurrence in children, whilst the reverse is true with respect to the chronic forms of the disease, and then remarking the various terminations of the disease either in resolution, or an increasing chronic discharge from the bronchial membrane, accompanied or not by hectic, in death occasioned by a sudden obstruction of a large tube, in the development of tubercle, in pneumonia, in death owing to an excessive secretion into the bronchial tubes, or in consequence of hydrothorax, he passes on to the consideration of—

CHRONIC PRIMARY BRONCHITIS—of which he says we may get a good idea by considering it a species of gleet of the mucous membrane, in which the inflammatory irritation, if it exists, is in many cases not so severe as to act sympathetically on the system—the various functions may go on during its presence; during the Summer season there is more or less complete remission of the symptoms, while on the approach of Winter the cough and the expectoration become more troublesome. In the course of some years the duration of the remissions becomes less, their completeness diminishes, and a permanent irritation and flux are established; the result of which may be dilatation of the tubes, emphysema, which affections may be accompanied with phthisis, disease of heart, hydrothorax, or general dropsy. When the flux is excessive, a great degree of emaciation is often observed, which may excite a suspicion that the patient is labouring under tubercular phthisis; the healthy state, however, of the circulating and digestive systems will serve to assist in the diagnosis. Our author here observes that the complication of chronic bronchitis with tubercular phthisis is much more common than is generally supposed, more particularly in individuals who have passed the meridian of life; the transition of the one affection into the other being marked by a general though gradual failure of the powers of life, by acceleration of the pulse, and a slow, though decided, emaciation of the patient. Under such circumstances a careful examination will detect some solidity in the upper portion of one lung.

Our author next points out the importance of attending to the secretions from the bronchial mucous membrane in relation to the symptoms, and studying their changes in connexion with the history of the case. These secretions from the bronchial membrane, when in a state of irritation, he divides as follows:—

*First.*—Transparent mucous secretions.

*Second.*—Opaque mucous, or albuminous secretions.

*a.*—Amorphous.

*b.*—Moulded to the form of the tubes.

*Third.*—Muco-puriform secretions.

*Fourth.*—Puriform secretions.

*Fifth.*—Serous secretions.

On these various forms of secretion the author makes some valuable practical remarks, some of which we shall present to our readers in as succinct a form as the importance of the subject will allow;—and first of the—

*Transparent Mucous Secretions.* This form is most commonly met in the earlier stages of acute bronchitis; it is generally preceded either by a dry cough, or by a cough with a serous expectoration. Both the quantity and tenacity of this secretion may vary in different cases: the latter quality is generally proportioned to the violence of the irritation. Transparent and tenacious mucus, however, is observed in other cases of bronchitis—it is sometimes seen to replace the muco-puriform secretion, which occasionally loses both its diffluent character and its opacity, and becomes difficult of expulsion. This change is generally accompanied with general constitutional disturbance. Dr. Stokes here is inclined to connect in some way the occurrence of this transparent and viscid secretion with a state of irritation, wherein the secretion does not relieve the morbid action. "We find," he says, "that the sooner the opaque sputa appear, the sooner shall we observe the convalescence of the patient; and that in those cases where this salutary change is delayed, the sufferings and danger are proportionally increased. How commonly do we observe this in phthisis, in which the bronchial irritation seems to continue in its first stage for an indefinite length of time, and in which there is every indication of a local but unrelaxed irritation of the lung: and in the cases of Laennec's emphysema, we may see other instances of a bronchial disease, which has not been relieved by the more elaborated secretion, and which consequently has continued so as to disorganize the lung."

*Opaque Mucous, or Albuminous Secretions.* With respect to these, they are generally preceded by the formation of the transparent secretion, occurring with, or without a symptomatic fever.

Of this species of expectoration our author notices two kinds, 1st. the amorphous variety, and next that in which the secreted matter adapts itself to the form of the bronchial tubes, so as to produce casts of the air-passages.

"In their ordinary form we find these sputa to consist of shapeless masses of a dull white colour, with a slight yellow tinge. These masses may be expectorated with scarcely any accompanying serous fluid, when they unite, more or less, so as to form a semi-fluid adhesive mass. In other cases, however, a considerable quantity of serous fluid is expelled along with them, when we observe them more frothy; and presenting the appearance of rounded sputa, floating in a nearly transparent fluid, of much less tenacity, and containing a few albuminous striæ, not unlike fragments of vermicelli."

With respect to the consistent secretion which is moulded to the form of the bronchial tube he observes that this formation of inspissated mucus, may be found either as a very circumscribed or a more general lesion. In the former of these a plug is formed, which by obstructing one of the larger tubes, may bring on violent dyspnœa, or even death; while in the second, cylinders of this substance, corresponding to the form of the bronchial tree, are found to follow its ramifications nearly as far as it is possible to trace the bronchial tube.

We next come to the *muco-puriform*, and *puriform secretions.* Our author considers them together; the former is of much more frequent occurrence than the latter, a completely purulent expectoration being an unusual occurrence in any pulmonary disease, even in those in which ulceration or suppuration has taken place. It is occasionally observed in the advanced stage of phthisis and pneumonia. If the bronchitis in its earlier stages has been neglected, or treated with timidity, the muco-purulent secretion in the advanced stages will be abundant, and may produce death by a mechanical obstruction.

In order, says our author, that the change from the mucous to the muco-puriform secretion may be considered a favorable indication, it is necessary that certain circumstances should attend it. These are, that the expectoration shall become easier, the pulse softer and slower, and the fever diminishing. With respect to the physical signs, the muco-crepitating râle becomes larger, the respiration returning from above downwards, the action of the heart becomes quiet, and the sound on percussion clear, even in the postero-inferior portions of the lung.

*Serous Secretions.* This form of secretion is met with in a great number of cases of bronchitis and phthisis, even where other and very different secretions are taking place from the lung. It is observed in the earlier stages of bronchial inflammation, before much mucus has been formed, and again in the advanced stages, when an opaque or muco-purulent matter is abundantly secreted, and lastly it occurs as the principal secretion, as is seen in many cases of *humid asthma,* in which it may be formed in great quantity. Andral mentions a case in which a sudden and very copious secretion of serous fluid from the lung coincided with the disappearance of an hydrothorax. Our author next proceeds to consider the

Physical Signs of Bronchitis, and first of the primary bronchitis, in its acute and chronic forms. These signs he considers in the following order:

*First.* The results of percussion.

*Second.* Signs discoverable by the sense of touch.

*Third.* Signs discoverable by auscultation.

With respect to the first class, namely those derived from percussion, he observes that in general there is no perceptible change in the clearness of the sound on percussing the thorax, there being but one case in which simple bronchitis is ever accompanied with decided dulness, namely, that in which a vast secretion of mucus, or muco-purulent fluid exists in the bronchial tubes—in those cases also wherein the areolar structure of the lung is the seat of disease, such as œdema, pneumonia, or tubercle, there is some dulness, the amount and site of which vary according to circumstances.

Dr. Stokes proposes a very ingenious mode of accounting for the absence of dulness in bronchitis, which he offers however merely as a conjecture; namely that pending the turgescence of the bronchial membrane, some dilatation of the air-cells takes place, so that the air thus accumulated compensates for that which has been displaced by the turgid state of the mucous membrane. Still, though percussion may give no direct result in bronchitis, its employment may be attended with important advantages in the particular diagnosis.

"Thus," says our author, "suppose that after the existence for three or four days, of fever, cough, hurried and difficult breathing, the chest still sounds well, the great probability is that the disease is bronchitis. The patient has had an acute inflammatory affection of the lung, and but of a few days standing: this must be either bronchitis, disease of the serous membrane, or of the parenchymatous structure. Here the absence of dulness is of the greatest importance: for were it a case of pleuritic effusion, or of disease of the substance of the lung, the great probability is, that by this time a degree of dulness would be manifested: in the one case the lung would be compressed, and its place occupied by a liquid effusion: in another, more or less obliteration of the air-cells would take place, from congestion, or inflammation. *The absence then of dulness, with the existence of acute irritation of the lung, which has continued for several days, forms an important argument that the case is one of uncomplicated bronchitis.*"

Our author next insists on the insufficiency of mere symptoms independent of physical phenomena to diagnose simple bronchitis, there being a vast number of examples of disease of different kinds, in which the symptoms are only those of bronchitis, or at least may be referred to this lesion. If however, on examining any particular case of this kind, we find a dulness on percussion, we may rest satisfied that something more than bronchitis exists. Dulness of sound from a copious effusion of mucus into the tubes can occur, according to our author's experience, only in the last stages of the disease. In one case however of mere bronchial disease, namely, in dilatation of the tubes with compression of the intermediate pulmonary tissue, dulness of sound may be observed.

We derive considerable assistance in diagnosing tubercular development from knowing that in simple bronchitis there is nothing to produce dulness of sound. The value of knowing this will be obvious, when we consider the great similarity in the symptoms between tubercular phthisis and bronchitis.

With respect to the signs discoverable by the sense of touch, there is not much to be said. In many cases, particularly after the first stage of the disease, a distinct vibratory sensation may be perceived on laying the hand on the thoracic parietes, which may be detected both during inspiration and expiration, more distinctly however in the former, and more evidently in the child and the female than the adult male. This vibration is also more distinct in the middle and inferior than in the upper portion of the lung. This vibration is not heard either in pneumonia or pleuritis. In the latter affection, however, a phenomenon may occur which may be confounded with it, namely the sensation of *friction of ascent and descent*, which is perceived in certain stages of dry pleuritis—the stethoscope however will enable the practitioner to distinguish between these, as in the majority of cases of pleuritic friction the respiratory murmur may be heard without any admixture of rhonchus.

The causes which modify the respiratory murmur in the passage of air during respiration are thus enumerated by our author:

*First.* The turgescence of the mucous membrane, a cause which principally affects the phenomena of the smaller tubes and air-cells.

*Second.* The existence of an anormal secretion into the cavity of the tube itself; and

*Third.* The existence of spasm, the amount of which varies in different individuals. It is these causes which form the numerous varieties and combinations of Laennec's *sonorous, sibilous,* and *mucous râles.* Our author states as a general rule that the modifications of sound connected with turgescence of the mucous membrane and spasm, are to be found principally in the first or dry stage, while those produced by the passage of air through fluid in the tubes, are most evident in the second or secretive stage:—and again, that in the acute stage, during ordinary respiration, the louder and more intense the râles are, the more severe is the disease. To this rule he makes one important exception first observed by him in bad catarrhal fevers, and one of great practical importance. In such cases, he says, during ordinary respiration, we may hear little or no râle, and yet the disease may be so violent as to threaten the patient's life. The reason which he assigns for this is, that the finer ramifications of the bronchial tubes are so turgid, that during ordinary respiration, the air does not enter them with sufficient force to produce a tone, while by making the patient take a forced inspiration, we are astonished at the intensity, number, and variety of the sounds produced. In such cases it generally happens, that as the patient gets better, the râle during ordinary respiration, becomes distinct and constant, an increase of râle during ordinary breathing pointing out a decrease of disease.

In some cases of chronic bronchitis attended with a muco-purulent secretion, and in which a râle exists which is almost crepitating, there may be a risk of confounding the disease with phthisis; the decided dulness however, whether general or partial, existing in phthisis will aid in the diagnosis—sometimes again a partial dulness may be met in both diseases; but here again we have a means of establishing the diagnosis; for in phthisis, even where the whole lung is tubercular, the dulness is almost always greatest in the upper parts of the lung, while in the case of bronchitis the reverse takes place, the dulness in this latter case being in the inferior lobes; which circumstance seems to be occasioned by the accumulation of mucus in the more depending portions.

The puerile respiration again may occur in both affections. When it occurs in bronchitis however, it is in the upper portions of the lung it is principally observed, whilst the reverse happens in phthisis.

An exacerbation sometimes occurs in intense bronchitis indicated by the opaque muco-puriform expectoration changing its character and becoming transparent and viscid, a change accompanied moreover with corresponding stethoscopic phenomena, the muco-crepitating râle becoming smaller and sharper and the extinction of the respiratory murmur becoming more complete. This is apt to be confounded with certain stages of pneumonia. The clearness, however, of the sound on percussion, and the absence of the bronchial respiration will assist in forming the diagnosis.

Our author now passes on to the consideration of the next physical sign accompanying bronchitis, and that is the complete suspension of the respiratory sound in certain portions of the lung. In general this phenomenon is only temporary; it may however be permanent; if the former, it will frequently disappear after a fit of coughing; which led Laennec to consider it as occasioned by the obstruction of mucus—it may however be caused by spasm. Hypertrophy of the bronchial mucous membrane also may not only modify but altogether annihilate the respiratory murmur, of which instances have been adduced by Andral and Reynaud. Our author before proceeding to some of the other varieties and results of bronchitis lays down the following propositions as containing the state of our knowledge with respect to the physical diagnosis of simple bronchitis.

“ 1st. That in almost all cases percussion gives no direct sign.

2d. That an accumulation of mucus in the inferior portion of the lung may give a certain degree of dulness.

3d. That in the great majority of cases in which there is a co-existence of the signs and symptoms of bronchitis with dulness, we may infer the existence of some disease, either of the parenchyma or the pleura.

4th. That conversely, the absence of dulness with the existence of irritation of the lung, gives a great probability that the case is one of simple bronchitis.

5th. That a copious effusion of muco-purulent matter may exist in the bronchial tubes, without perceptible dulness of sound on percussion.

6th. That in certain cases of bronchitis with effusion a metallic sound may be produced on percussion. This is somewhat similar to the *bruit de pot fêlè* of caverns, but is to be distinguished from it by the clearness of sound, its greater diffusion, and the absence of the stethoscopic signs of a cavity.

7th. That in many cases, on application of the hand, a distinct vibration is felt in accordance with the motions of respiration.

8th. That the modifications of respiration, as observed by the stethoscope in bronchitis, seem to be connected with mechanical obstruction more or less complete, and which may proceed from one or all of the following causes:—turgescence or hypertrophy of the mucous membrane, the existence of various secretions, and, lastly, the occurrence of spasm.

9th. That in the mode of occurrence of the various phenomena there are the greatest possible differences in different individuals.

10th. That as a general rule it may be stated, that the more intense the sonorous, sibilous, or mucous râles, or any combination of them, be during ordinary respiration, the more severe may the disease be considered.

11th. But that in certain cases of intense bronchitis of the minuter tubes, the sounds during ordinary respiration cease to be a measure of the intensity of disease, as they become louder during the convalescence of the patient.

12th. That in the secretive stage of bronchitis the mucous rattle may occur, on the one hand, with large and isolated bubbles, and, on the other, may pass into a râle almost crepitating, the sound on percussion still continuing clear.

13th. That in consequence of bronchial inflammation the entrance of air into a certain portion of the lung may be prevented, under which circumstances the signs are nullity of respiration, with persistence of clearness of sound.

14th. That this obstruction may result from an organic change of the mucous membrane, or from the plugging up of the tubes by their own secretion.

15th. That in the first of these cases the absence or diminution of the respiratory murmur is permanent, while in the second it may be temporary, and removable by a fit of coughing; yet even in this case the obstruction by a concrete mucus has continued from the period of its occurrence until the fatal termination.

16th. That if in a case of mucous catarrh a sudden dyspnœa supervenes, with absence or diminution of the respiratory murmur in a particular portion of the lung, this portion also preserving its clearness of sound on percussion, we may make the diagnosis of obstruction of the bronchial tube by its own secretion."

ACUTE SECONDARY BRONCHITIS. Our author having thus far considered that form of bronchial disease, in which the affection of the mucous membrane appears to be the first link in the chain of morbid action, and the fever sympathetic, next proceeds to consider the disease in its secondary form, when it seems to be the result of an influence which seems to act on the whole system, or to proceed from a sympathetic irritation, the consequence of local disease in some other system. He now proceeds to examine, first, into the bronchitis of typhus fever; next into that of the exanthematous diseases; and lastly, he makes some observations on those forms of bronchitis, which in other specific contaminations of the system, and which may be called chronic secondary catarrhs, and the sympathetic coughs arising from irritation of the digestive system.

*Bronchitis of Typhus.* In some cases of typhus the lungs may be severely attacked, and fatal asphyxia occasioned from excessive secretion from the bronchial membrane; this may occur under two circumstances, the one where the symptoms are manifest, the other where the disease is latent. In those cases wherein the bronchitis assumes a prominent and formidable character, we have, in addition to the other phenomena of fever, lividity of countenance, cough, hurried breathing, and expectoration—and even though these symptoms be but faintly marked, still a bronchitis may exist of the intensity and extent of which nothing but a physical examination can convince us. In the fevers of this country this bronchial affection is found to coexist with gastro-enteric inflammation—in some cases the disease predominates in the respiratory, in others in the digestive system. This predominance of disease also often alternates between the thoracic and abdominal cavities. Our author here makes a remark of great practical importance by stating, that with respect to the pathology of mucous membranes in fever, although the gastro-intestinal mucous surface may be, and often is affected, while but little, if any, disease exists in the respiratory organs; yet that the converse of this proposition is seldom true, a point of the utmost importance in practical medicine, as bearing on the application of general, local, and specific treatment. Our author now points out certain differences between the bronchitis of typhus and the primary species. The *first* and most important is the want of proportion between the intensity of the phenomena and the sufferings of the patient; the former being extreme, while the latter, at least until the last stage, are comparatively trifling. The *second* point of difference, and one closely connected with the former, is, that the intensity of the râle during ordinary breathing is, in many cases, not a measure of the violence of the inflammation or congestion of the air-tubes, as during ordinary breathing the râles may be but slight and diffused, and yet on a forced respiration they may become most intense. This Dr. Stokes explains by the great obstruction of the minute tubes coupled with the great debility of the patient. As the disease declines, we have a loud râle during ordinary respiration; the increase of râle thus indicating a decrease of the disease.

The *third* point of difference is, that the character of the râle in bronchitis accompanying typhus would incline one to conclude that the disease more especially affects the large tubes, where the lining membrane has the proper characters of a mucous structure.

Lastly, in the severe bronchitis of typhus the morbid phenomena predominate chiefly in the lower and posterior parts of the lung.

The author next proceeds to consider bronchitis in relation to the exanthematous diseases. After first insisting on these diseases being instances of specific fevers, in which the cutaneous irritation is but a single link in the chain of morbid action, and not even the first link, as the viscera, in the majority of cases, appear to be the first to suffer, he shews that the exanthemata have certain points of resemblance to typhus, namely, in the circumstance of periodicity, and in the local affections characterising them not being primary, constant, or proportioned to the general symptoms. There is however one mark of difference between the two affections, which is, that the secondary irritations in the exanthemata are more violent and more constant than in typhus, and that the inflammations accompanying them, at least in measles and scarlatina, have more of the sthenic character. Another difference, and that an anatomical one, exists; which is, that in the exanthemata there is a greater chance of the serous membranes being involved than in typhus. After stating that irritations of the pulmonary system are more commonly met with in measles and scarlatina, and those of the digestive system in variola and erysipelas, he next proposes the question, whether the inflammation of the mucous membrane in the former case is specifically different from that in idiopathic bronchitis. To a satisfactory solution of this question sufficient observations are yet wanting. The author next proceeds to the consideration of—

CHRONIC SECONDARY BRONCHITIS, and points out the most striking instances of bronchial irritation connected with those slower actions which result from chronic constitutional disease, such as gout, scrofula, syphilis, &c., which, as well as those affections of the system called fevers, may, and actually do, produce their specific forms of bronchial inflammation. Numerous instances may be adduced to prove that the pulmonary system may be affected, either primitively or consecutively, by the gouty irritation. The syphilitic poison also has the power of exciting all the symptoms of bronchial irritation and inflammation, which may assume either an acute or chronic form; in the first instance it is analogous to the bronchial irritations of the exanthemata, while, in the second, there is a chronic irritation, which, when combined with the syphilitic hectic, and with periostitis of the chest, closely resembles true pulmonary phthisis. In support of the opinion that the syphilitic poison may affect the pulmonary, as well as the osseous, cutaneous, and mucous tissues, Dr. Stokes adduces the high testimony of his friend and colleague, Dr. Graves. The diagnosis he lays down between this syphilitic irritation of the bronchi and tubercle, is "the want of accordance between the physical signs and the constitutional symptoms." He next proceeds to consider—

*Sympathetic Cough*, of which there may be two forms, the primary irritation in both residing in the digestive system. The first form is, the cough resulting from gastric inflammation, and the second that from intestinal worms. The following characters of this gastric cough are taken from Broussais, to whom our author acknowledges himself much indebted for valuable information on this subject. "It comes on with violent shocks, which occur at each inspiration, and those violent paroxysms, which would produce swelling, and lividity of the countenance, are never observed. It is more alleviated by cooling and slightly acidulated drinks than by bleeding; and lastly, with reference to the expectoration, it may be present or absent according to the degree of the bronchial irritation, but its excretion may be suspended by means calculated to relieve gastritis, and this suspension is advantageous to the patient."

Dr. Stokes now proceeds to state the results of his own experience on this interesting subject of sympathetic affections. These he is inclined to consider at first only lesions of function, but that when they are violent or long continued, they become complicated with organic and structural change.

"We may," he says, "see a patient with the most aggravated cough, yet with a chest clear on percussion, and the murmur either pure, or mixed here and there with a little sonorous or mucous rattle. This want of proportion between the physical signs and the functional lesion leads us at once to the principle of diagnosis, what may be announced to be, *that when distressing pectoral symptoms exist, the morbid physical signs being either absent, or if present, yet revealing an amount of disease too slight to account for the symptoms, we may make the diagnosis of sympathetic irritation.*"

Our author now directs his attention to the sympathetic cough arising from the irritation of intestinal worms. The cause of this cough is often difficult to be ascertained. He here enumerates certain circumstances which should lead us to suspect its real source.

*First.* Its character; the cough, whether it be laryngeal or pulmonary, being generally spasmodic, often violent, and almost always dry.

*Second.* The absence of physical signs of pulmonary disease; or if they be present, their want of proportion to the symptoms. In this investigation both the active and passive signs must be carefully examined.

*Third.* The absence of symptoms of laryngitis, or organic disease in the vicinity of the trachea.

*Fourth.* The healthy state of the pharynx.

*Fifth.* The failure of treatment directed to the chest, whether of an antiphlogistic or antispasmodic nature.

Such a combination in a young person, more particularly in one in whom, from other considerations, one might be disposed to suspect worms, should incline very much to the belief in their existence. We now come to the author's—

*Treatment of Bronchitis;* and first of the simple and mild form, which is commonly met with about the period of the first dentition of children. In the apyrexial form of this disease he recommends a free and complete division of the gums, the incision being proportioned to the number of teeth likely to appear. Attention to the bowels by the use of hydrargyrus c̄ creta and rhubarb, followed by castor oil and minute doses of hippo, include all the treatment necessary. When there is violent fever, however, bloodletting both general and local are found necessary, when the disease occurs in a strong child, which has passed the age of a year. The remainder of the treatment consists in the use of internal remedies, the principal of which are the potassio-tartrate of antimony, and the combination of calomel and hippo. In simple bronchitis, where the inflammatory symptoms run high, our author gives the decided preference to the former.

*Treatment of Bronchitis in the Adult.* Should the accompanying fever be inflammatory the lancet must be employed. The author here makes an observation of considerable importance in a practical point of view, namely, the great difference in the result of bleeding in serous and parenchymatous inflammations, and that in mucous inflammation; experience shews that general bleeding has much more influence in cutting short the former than the latter diseases. He next points out the necessity of emptying the patient's bowels. The local detraction of blood is of prime importance after general bleeding. Local depletion is found more efficacious when exercised over the upper than the lower part of the chest. Certain circumstances, such as suppression of expectoration when coinciding with increase of fever; increased dyspnœa, when not produced by over-secretion; local dulness, arising from congestion of the substance of the lung—may render it necessary to repeat the local abstraction of blood even in an advanced stage of the disease. Of the internal remedies to be employed in bronchitis the solution of tartar-emetic holds the first place. Great caution however is necessary in its employment, should there exist symptoms of gastro-intestinal irritation. Our author follows pretty nearly Laennec's mode of administering this medicine.

In discussing the *Treatment of the Second Stage of Bronchitis,* the author lays down a pathological principle, which he considers of great importance in the practice of medicine, one however which we think has been practically acknowledged, and virtually if not expressly recognized from a very early period, namely, that in a vast number of general and local diseases two stages are observed, in the first of which an antiphlogistic treatment is necessary, and stimulation is injurious; whilst in the second, antiphlogistic is insufficient, and often injurious, while stimulation becomes necessary. Dr. Stokes considers the overlooking of this second stage as one of the greatest errors of the physiological school. This principle he now applies to the treatment of bronchitis in its second stage, and first he considers the employment of counter-irritation, which is inapplicable in the earlier periods of the disease, so long as the skin is hot, the pulse strong, the expectoration scanty and difficult. The author here very justly reprobates the mischievous practice adopted by routine practitioners of having recourse to blisters in the early periods of disease, even before any antiphlogistic means have been employed for the purpose of reducing the general febrile state. In selecting the place for the blister we should be guided principally by the physical signs, and in particular by the active and passive auscultatory phenomena. In cases where the minute tubes have been affected, where the convalescence is slow and doubtful, and where there are alternations of hectic and synocha, the use of the seton has been found beneficial.

With respect to stimulating remedies in this stage the Doctor gives the preference to the decoction of polygala combined with carbonate of ammonia and the camphorated tincture of opium and squill. Next in point of efficacy he ranks the balsams, and the preparations of gum ammoniac, myrrh, and squill. The inhalation of balsamic preparations he considers attended with no inconsiderable degree of danger, as he has known several cases where a chronic bronchitis was converted into an acute, and as might be expected, fatal pneumonia, by their use. The bad consequences of the misapplication of this class of remedies may be best avoided by attending to the following circumstances:

*First.* To provide that the stimulating remedy shall be preceded by a fit antiphlogistic treatment.

*Second.* To combine it with a revulsive plan, such as blisters, cupping, warm bathing, &c.

*Third.* To omit the remedy on the slightest appearance of new irritation, either in the affected part, or in any other vital organ.

Our author next makes some remarks on the treatment of the apyrexial form of chronic primary bronchitis—in such case where the disease has not lasted more than a year or two, and in which the Summer remission has been complete or nearly so, we may hope to be able to do good; and if there be no evidence of tubercle, dilated tubes or enlarged air-cells, the prognosis will be still more favorable. If however the disease has been of several years standing, and the Summer remission has been very slight, and if there be permanent dyspnœa, palliation, not cure, is all we can expect. On the therapeutic means to be employed in this affection the author makes some practical remarks which we shall here present to the reader: 1st. he considers the employment of revulsives calculated to produce the very best effects. 2d. Sponging a large surface of the chest daily with a liniment consisting of spirit of turpentine and acetic acid, so as to keep out an erythematous state of the skin, he has found extremely beneficial, not only in bronchitis but even in confirmed phthisis. And the benefit to be derived from this remedy he thinks should be referred not merely to its counter-irritating properties, but to the absorption of the ingredients by the surface, by which they act on the mucous surface as direct stimuli. Another therapeutic remedy from which the Doctor has derived advantage is the inhalation of aqueous vapour impregnated with a narcotic. Twelve or fifteen grains of the extract of hemlock may be diffused in a proper inhaling apparatus, and the vapour drawn into the lungs for a quarter of an hour once or twice a day.

Of internal stimulants he considers the terebinthinate preparation to hold the first place. The various balsams, exhibited either alone, or in combination with a sedative and tonic, are often found to act well. The late Dr. Armstrong, if we remember rightly, was a great advocate for copaiba in chronic affections of the bronchial mucous membrane. From the efficacy of strychnine in the analogous affection of the gastric mucous membrane, the author conceives that there may be good reason to hope, that in the pulmonary disease it might also prove useful—quinine and the preparations of iron, especially when the powers of life are low, and antiphlogistic measures have preceded, are of acknowledged utility—where the flux is excessive, astringents are indicated, and the astringent remedy most approved by the author is the acetate of lead. But in cases of over-secretion, unless the patient be much debilitated either from age or disease, the emetic plan he thinks is always to be preferred to the astringent. Emetics he conceives to produce very beneficial effects, not only by getting rid of the super-secretion mechanically, but also by exerting a favourable action on the cause of its production. He here adduces the testimony of Dr. Giovanni de Vittis in favor of emetics in this disease. The last point of treatment to which the author alludes is the use of sedatives and narcotics. These are chiefly useful, when the cough is severe and the expectoration not abundant. The preparations of opium, hyosciamus, or conium, and the combination of these with a small portion of belladonna, he has found extremely useful. He next comes to consider the

*Treatment of Secondary Bronchitis*—more particularly as occurring in typhus. Though the principles of treatment in this form are the same as in the idiopathic disease, yet in their application to practice, he conceives that certain variations are to be attended to. These he states as follows :—*First*, the antiphlogistic treatment is not to be employed so boldly nor so long.—*Second*, the stimulating treatment may be resorted to at an earlier period, and with much greater boldness. *Third*, that the use of blisters may be employed also at an earlier period. The other directions given by the author for the treatment of bronchitis in typhus with respect to the use of the lancet, etc, we abstain from giving, there being nothing very novel in them.

From the length to which we perceive our analysis of this work has already run, we find it necessary to defer further notice of it till our next number. We cannot however lay down our pen without expressing our deep sense of acknowledgment to Dr. Stokes for this so splendid a contribution to thoracic pathology. The masterly and practical views taken by him of this so important a class of diseases, diseases too, to which, in some form or other, so vast a number of the inhabitants of these countries are daily falling victims, cannot fail to place him in the very first rank as a zealous pathologist, and a sound practical physician. He has indeed in producing this work more than realized the hopes which we formed of him from his numerous contributions to medical science communicated by him through the several medical journals of the day.

